# Understanding genetic variability: exploring large-scale copy number variants through non-invasive prenatal testing in European populations

**DOI:** 10.1186/s12864-024-10267-5

**Published:** 2024-04-15

**Authors:** Zuzana Holesova, Ondrej Pös, Juraj Gazdarica, Marcel Kucharik, Jaroslav Budis, Michaela Hyblova, Gabriel Minarik, Tomas Szemes

**Affiliations:** 1 Geneton Ltd, Bratislava, Slovakia; 2 Comenius University Science Park, Bratislava, Slovakia; 3 Slovak Centre of Scientific and Technical Information, Bratislava, Slovakia; 4 Department of Molecular Biology, Faculty of Natural Sciences, Comenius University, Bratislava, Slovakia; 5 TRISOMYtest Ltd, Nitra, Slovakia; 6 Medirex Group Academy, Nitra, Slovakia

**Keywords:** Copy number variation, Whole genome sequencing, Non-invasive prenatal testing, Population study, Large-scale CNV frequency comparison

## Abstract

Large-scale copy number variants (CNVs) are structural alterations in the genome that involve the duplication or deletion of DNA segments, contributing to genetic diversity and playing a crucial role in the evolution and development of various diseases and disorders, as they can lead to the dosage imbalance of one or more genes. Massively parallel sequencing (MPS) has revolutionized the field of genetic analysis and contributed significantly to routine clinical diagnosis and screening. It offers a precise method for detecting CNVs with exceptional accuracy. In this context, a non-invasive prenatal test (NIPT) based on the sequencing of cell-free DNA (cfDNA) from pregnant women’s plasma using a low-coverage whole genome MPS (WGS) approach represents a valuable source for population studies. Here, we analyzed genomic data of 12,732 pregnant women from the Slovak (9,230), Czech (1,583), and Hungarian (1,919) populations. We identified 5,062 CNVs ranging from 200 kbp and described their basic characteristics and differences between the subject populations. Our results suggest that re-analysis of sequencing data from routine WGS assays has the potential to obtain large-scale CNV population frequencies, which are not well known and may provide valuable information to support the classification and interpretation of this type of genetic variation. Furthermore, this could contribute to expanding knowledge about the central European genome without investing in additional laboratory work, as NIPTs are a relatively widely used screening method.

**Supplementary Information:**

The online version contains supplementary material available at 10.1186/s12864-024-10267-5.

## Introduction

Cell-free DNA (cfDNA) is released from cells into the circulatory system and can be found in various body fluids, including plasma, cerebral spinal fluid, pleural fluid, urine, and saliva. In certain conditions like pregnancy, organ transplantation, and cancers, additional DNA can be released into circulation from affected tissues. Detection of cfDNA in peripheral blood can identify abnormalities noninvasively, making it valuable for various applications such as noninvasive prenatal testing (NIPT) or cancer diagnosis [1]. Prenatal testing has undergone a prolonged development from the traditional invasive methods such as amniocentesis or chorionic villus sampling [2]. Since the discovery of cell-free placental DNA (cfpDNA) in maternal plasma, non-invasive prenatal testing (NIPT) has been integrated into clinical practice. It has become a standard practice in developed countries. In some countries, these tests are already implemented in public prenatal care. In the Netherlands, NIPT became available in 2014 as part of the TRIDENT-1 study for pregnant women at increased risk of common trisomies [3]. Subsequently, the TRIDENT-2 study was launched in 2017 to offer NIPT as the first-tier test for all pregnant women [4].

Most of the current NIPT approaches are based on low-coverage whole genome sequencing (WGS) of DNA from the blood plasma of pregnant women. In this way, a chromosomal ploidy can be determined [5], and the technique proved to bring reliable results in detecting trisomies and other fetal chromosomal abnormalities [4, 6]. However, several extensions also allow the detection of subchromosomal aberrations, such as microdeletions and microduplications [7]. This type of genetic variation, also known as copy number variants (CNVs), results from the loss or amplification of DNA segments ranging from 50 bp to tens of Mb. It has previously been shown to be a common part of the human genome [8, 9] and participates in population diversity [10, 11]. Furthermore, CNVs play an important role in evolution, contributing to the development of various diseases, influencing different biological processes that affect morphological variability, and affecting the host-microbiome interaction or susceptibility to infection [12].

Clinical tests such as NIPT are primarily focused on the genetic analysis of the fetus. However, maternal DNA is also analyzed, which offers additional data for further supporting analyses. Individuals who have undergone NIPT represent a minimally limited sample (women of reproductive age) but still a relatively large sample of the adult female population. Therefore, sequencing data could be a valuable source for population studies. This proposal is based on our previous work, where we proposed NIPT as a source of population-specific allelic frequencies [13], and on subsequent work where the potential of CNV ≥ 600 kbp in the Slovak female population was shown [14]. This study focused on comparing even smaller variants, CNV ≥ 200 kbp, in pregnant women from Slovakia, Hungary, and Czechia. We demonstrated that without additional financial investments in laboratory preparations, this approach provides the potential to obtain the population frequencies of large-scale CNVs. Our research broadens the general knowledge of this type of human genetic variability, which is currently poorly studied. Consequently, maternal genomic data obtained from NIPT can offer valuable information for researchers, laboratory diagnosticians, and clinical genetics since this knowledge could be used as supporting evidence for classifying and interpreting other variant findings.

## Materials and methods

### Cohort specification

We have analyzed sequencing data of 12,732 women undergoing NIPT after the tenth week of pregnancy. The data were provided by TRISOMYtest Ltd., which is responsible for sample processing and sequencing analysis. Enrolled individuals are representatives of Slovak (9,230), Czech (1,583), and Hungarian (1,919) populations. The median age of the cohort is 35, ranging from 18 to 51 years. Data were collected between 2016 and 2021. All samples were processed using the same protocol and equipment type, though not necessarily in the same laboratory.

### Sample preparation

Plasma samples of pregnant women were collected and processed for analysis by the protocol described in our previous work [15]. Low coverage whole genome sequencing (0.3 ×) was performed by the Illumina NextSeq 500/550 platform as a part of routine NIPT. It was suggested that sample handling and data analysis contributed significantly to the previously reported excess of population-stratified variants [16]. Thus, we eliminated heterogeneity in sample processing between laboratories as much as possible, and only samples processed by the same protocol and sequenced on the same type of equipment were included in the following analyses.

The samples were anonymized prior to further analysis. The anonymization process involved assigning a number to each sample and discarding all other metadata.

### CNV identification

Sequencing reads were aligned to the reference genome GRCh37 using the Bowtie2 algorithm [17]. We used only information for the initial position of the mapped reads, while only reads with mapping quality ≥ 40 have been stored.

Then, GenomeScreen, a low-coverage, whole-genome NGS-based CNV detection method [18] (validated in our laboratory and currently available commercially), was used to identify CNVs. Reads were grouped into bins with a size of 20 kbp. Then, a two-step normalization was employed: (1) LOESS-based correction to eliminate GC-bias [19] and (2) PCA normalization to remove higher-order population artifacts on autosomes [20]. To enhance result accuracy, we filtered out regions prone to errors, particularly those with variable or low mappability. These regions are predominantly located near centromeres or chromosome ends. Finally, the genome coverage signal was split into regions with equal levels using the circular binary segmentation algorithm from the R package DNAcopy [21], and segments with abnormal copy numbers were identified. Due to the detection capability of the methodology used, the lower limit for the identification of maternal CNVs was set to 200 kbp, considering only segments with at least 60% signal increase/decrease compared to the referencebased on the findings in [18]. Unlike the study in [18], we analyzed mixed samples of maternal and placental DNA, with a low proportion of placental DNA (approximately 10%; samples with high placental DNA content were excluded). Consequently, these mixed samples exhibit behaviour similar to pure samples. To ensure accuracy, we increased the detection threshold to 200 kbp, compared to the 100 kbp threshold deemed feasible in [18] with the same setup.

CNVs were categorized into groups based on proximity; a CNV was placed in a group if its start and/or end coordinates differed by less than two unfiltered bins (40,000 bp) from another CNV in the same group. Although this approach theoretically allows for non-overlapping CNVs in the same group, it never happens in practice. Then, we assigned the same CNVs from different populations to each other and determined whether there was a significant difference in their representation between populations.

### Statistical analysis

Python library pandas were used for data analysis [22]. The significance of our findings was evaluated using statistical tests implemented in the Python SciPy package [23]. Charts were created using the Python Plotly graphing library (Inc., P.T., 2015. Collaborative data science. Available at: https://plot.ly). The Chi-square test was used to determine the significance of differences between populations for all the following statistical analyses, including numbers, distributions, and overlaps of CNVs.

## Results

Our CNV calling pipeline has identified 5,062 CNVs ranging from 200 kbp to 75,260 kbp (median size 320 kbp). Altogether, 4,042 individuals (31.19%) present variation, of which 79.56% carried only one CNV, and 17.42% were carriers of at least two CNVs. Moreover, one woman from the Slovak population has shown a presence of even 32 CNVs, suggesting genomic instability. The gains-to-losses ratio was approximately 2.5:1 in all the populations (Table 1).

**Table 1:** Data summary for individual populations of pregnant women undergoing NIPT analysis

Population	Samples	Samples with at least 1 CNV	CNVs	Gains	Losses
Slovak	9 230	2 900	3 585	2 578 (72%)	1 007 (28%)
Czech	1 583	510	622	460 (74%)	162 (26%)
Hungarian	1 919	632	855	611 (71%)	244 (29%)
Sum	12 732	4 042	5 062	3 649 (72%)	1 413 (28%)

**Fig. 1:**
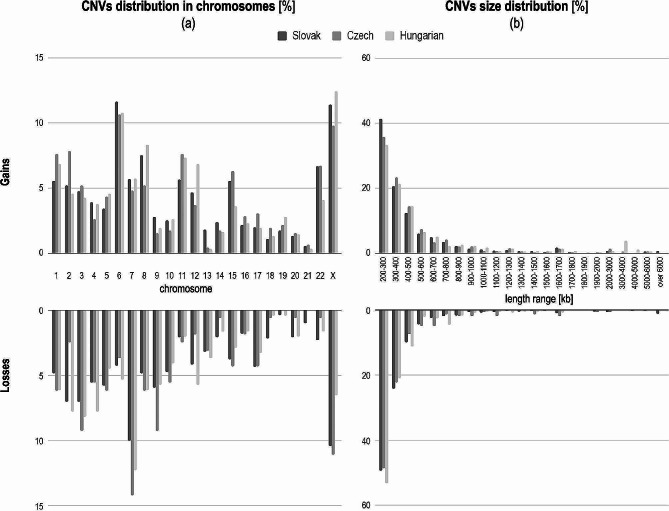
Characteristics of maternal CNVs identified in all the populations. (**a**) Distribution of gains and losses on individual chromosomes and (**b**) according to size ranging from 200 kbp to ≥ 6,000 kbp

Excluding the sex chromosome X, the sixth chromosome contained the most gains, precisely 11.6%, 10.7%, and 10.8% of all found gains, in the Slovak, Czech, and Hungarian populations, respectively. On the other hand, the highest count of losses was observed on chromosome seven for all three populations (Slovak 10.0%, Czech 14.2%, and Hungarian 12.3%). With a few exceptions, the overall count of CNVs decreased with the length of the chromosomes (Fig. 1a, Supplementary Table 1). In order to find out the length distribution of the variants, we divided them into size ranges of 100 kbp. The most frequent size of CNVs was 200 kbp to 500 kbp; this range contained around 70–85% of all the CNVs. Larger CNVs were rare, and their count decreased with the increasing size (Fig. 1b, Supplementary Table 2).

**Fig. 2:**
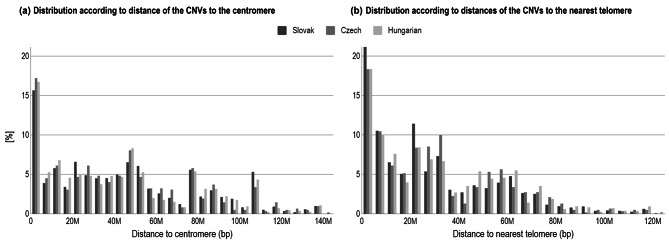
Relative frequency histogram of CNV distances to (**a**) centromere and (**b**) nearest telomere, respectively

By comparing the distributions of CNV distances, either to chromosomal ends or centromeric regions, we found CNVs overrepresented close to telomeres and centromeres (Fig. 2). The average frequency of CNVs per one Mbp of random genome sequence was 0.041%, while the average CNV frequencies within 1 Mbp proximal to the centromere and telomeres were 8.48% and 7.70%, respectively (Table 2). However, CNVs are known to predominantly arise in these regions [24]. Additionally, the technical accuracy of the CNV detection method is lower in these areas due to typically low mappability. This technical limitation should not affect maternal CNV detection, given the significant signal difference between maternal and unaffected CNVs.

**Table 2:** Average CNV frequency within 1 Mbp of different genomic regions

Region	Slovak	Czech	Hungarian	Average
1 Mbp of random haploid genome sequence	0.040%	0.038%	0.044%	0.041%
1 Mbp proximal to centromere	5.76%	7.04%	12.66%	8.48%
1 Mbp proximal to telomeres	7.31%	7.41%	8.39%	7.70%

CNVs divided into groups based on proximity with allelic frequency over 1% (7 gains, 8 losses) is shown in Supplementary Tables 3 and 4. Using the Chi-square test, we compared population differences in the count of CNVs on all chromosomes; we found a statistically significant difference in the CNVs gains (*p*-value = 0.0113). A comparison of the individual population pairs showed a significant difference between Slovak and Hungarian populations (*p*-value = 0.0396 from the Chi-square test). However, when comparing population differences in the count of CNVs on individual chromosomes, we did not find any significant difference after the Bonferroni correction (0.05/23 = 0.0022).

We found a statistically significant difference in CNV length distribution between populations (*p*-value = 8.69 × 10^− 14^) when we compared the count of CNV gains in individual length ranges (Fig. 1b). The individual population pairs comparison showed a significant difference between Slovak and Hungarian populations (*p*-value = 8.88 × 10^− 16^). When we compared population differences in each individual population CNVs length range pairs, we found a significant difference between Slovak and Hungarian populations in length range 200–300 kbp (*p*-value = 0.000315), 3–4 Mbp (*p*-value = 1.86 × 10^− 18^) and 4–5 Mbp (p-value = 0.000225) and Czech and Hungarian population in length range 3–4 Mbp (*p*-value = 0.000758), all after Bonferroni correction (0.05/23 = 0.002). We did not find any significant population difference in the count of CNV losses in all individual length ranges.

We continued by searching the most prevalent CNVs in the population, specifically those with a frequency exceeding 1%, that can be considered copy number polymorphisms [25]. We found 7 gains and 8 losses, which showed allelic frequency ≥ 1% in at least one population (Supplementary Fig. 2, Supplementary Table 3). When we compared these variants with publicly available database gnomAD SVs v2.1 (European) [26], we found no comparable range in four cases (gains: 8:2,340,000–2,580,000; 15:32,020,000–32,420,000; 22:22,280,000–22,580,000; losses: 7:64,680,000–64,900,000; (Supplementary Table 4). After applying automated ACMG guidelines available at https://genovisio.com, 8 variants were classified as variants of uncertain significance (VUS) without known clinical relevance, and 7 variants were benign. According to the ISV tool [27], 4 variants were VUS, and 11 were benign. Using the artificial intelligence integrated into the X-CNV predictive tool [28], we identified 10 variants as benign, 2 as likely benign, 2 as VUS, and 1 as pathogenic. For 7 variants, prediction matched in all three tools (Supplementary Table 4).

Considering the counts of variants between populations, we found a difference in the representation of variants 8:2,260,000–2,640,000 (*p* = 2.18 × 10^− 8^), 8:2,340,000–2,580,000 (*p* = 2.29 × 10^− 13^), and 12:20,960,000–21,400,000 (*p* = 1.63 × 10^− 3^; statistically significant after Bonferroni correction; Supplementary Table 3). These CNVs were not present in at least one population, so we considered their occurrence zero in the given population. When comparing such CNVs only between the two populations with non-zero counts, we observed a different representation of 12:20,960,000–21,400,000 (SK-HU, *p* = 0.00168) (Supplementary Table 3).

Since CNVs can overlap different genomic regions, we explore the representation of protein-coding genes, long non-coding RNAs (lncRNA), and microRNAs (miRNAs) in our cohorts. Coordinates for individual genomic regions, known as biotypes, were obtained from the GRCh37 [29]. The ratio of CNV-biotype for gains and losses overlaps in the studied populations is shown in Table 3.

**Table 3:** The ratio of CNV-biotype overlaps in a given population for gains and losses

Biotype	Gains			Losses		
	Slovak	Czech	Hungarian	Slovak	Czech	Hungarian
gene*	30.79%	34.49%	26.77%	10.91%	7.39%	6.00%
lncRNA	36.30%	34.18%	26.13%	17.03%	18.84%	14.77%
miRNA	0.017%	0.016%	0.017%	0.006%	0.005%	0.004%

*gene represents protein-coding sequences, including both exons and introns; **lncRNA** - long non-coding RNA; **miRNA** - microRNA.

On average, 39% of CNV sequences overlap protein-coding genes, while 31% fall on gains and 8% on losses. Moreover, almost half of all CNV sequences (aver. 49%) overlapped lncRNA (32% of gains, 17% of losses). On the other hand, CNV-miRNA overlaps were near zero since miRNAs constitute a small portion of the genome. Every type of CNV-biotype overlap calculated separately is listed in Supplementary Tables 5 and plotted in Supplementary Fig. 1.

## Discussion

The MPS method has become an integral part of prenatal care in recent years, as it allows for non-invasive prenatal screening of fetal aneuploidies and structural aberrations. However, clinical assays such as NIPT are mostly single-purpose and focused on fetal genetic analysis. However, this approach provides a wealth of data from maternal DNA that can be used for other supporting analyses. Here, we propose additional possibilities for the use of genomic data generated by routine NIPT screening based on cfDNA sequencing from the plasma of pregnant women using a WGS approach.

Patients undergoing NIPT represent a population sample, so their genomic data can be valuable for population studies. This is particularly relevant in countries where NIPT has been implemented in public prenatal care, such as the Netherlands and Belgium [30]. On the samples of pregnant Slovak, Czech, and Hungarian women, we have shown that without additional investment in laboratory consumables, NIPT has the potential to obtain population frequencies of large-scale CNVs. Our findings could help to understand this important type of human genetic variability, as it is a poorly studied genetic phenomenon.

A negative correlation between the length and the number of CNVs in all populations is consistent with previous studies [14, 16]. Since shorter CNVs are less likely to hit a critical region, they are not subjected to such a substantial selection as large-scale CNVs. Losses are also known to be more deleterious to the genome than the CNV gains [31]. Accordingly, the overall gain/loss ratio was in favor of gains in all the populations. Although large-scale CNVs are common in normal individuals, the length and the type (gain/loss) of aberrations seem to be one of the most limiting factors reflecting the deleterious effect of CNVs on the viability of individuals.

CNVs were not uniformly distributed on chromosomes between populations (Chi-square test *p* = 0.0031) with depletion of losses on chromosome 2 in Czech samples (Chi-square test *p* = 0.042). However, since we tested multiple hypotheses (23 chromosomes), the difference was not significant after Bonferroni adjustment (Bonferroni-corrected *p* = 0.0022). On the other hand, the distribution of CNVs on chromosomes differs when compared with a previous study evaluating CNVs ≥ 600 kbp [14], suggesting that CNVs of different lengths preferentially occupy specific chromosomes. This could be related to gene density and type of genomic elements, as they are expected to be under different degrees of constraint for variation in copy number [10, 32]. The overall distribution of CNVs was not uniform through the chromosomes, but CNVs were enriched in telomere and centromere proximal regions. These findings support the previous studies showing CNVs near centromeres and telomeres more frequently than expected by chance [24, 33]. The length distribution of large-scale gains also differs between populations, while the Hungarians have shown to be the most different in our cohorts.

We found copy number polymorphism (defined as a variant with allelic frequency ≥ 1%) [25], which seems to be a Slovak population-specific gain of 8:2,260,000–2,640,000 (Supplementary Table 3). Although the CNV overlaps no protein-coding genes and was predicted to be benign, it spans 59 regulatory elements and 7 lncRNA sequences with potential biological functions. The loss of chr15:22,760,000–23,080,000 was frequent CNV overlapping a morbid gene *NIPA1* associated with hereditary spastic paraplegia. It was shown that the *NIPA1* inhibits bone morphogenic protein signaling, which is critical for regulating synaptic growth and axonal microtubules [34]. Thus, *NIPA1* loss-of-function may lead to defects in synapse and axon development [35].

We have shown that most individuals are carriers of one CNV ≥ 200 kbp, but a woman with 32 CNV findings was also present in our cohort. Such numerous large-scale CNVs suggest genomic instability that is often associated with cancer pathologies. After requesting the patient’s metadata from the laboratory, we found out that the patient was suspected of colorectal carcinoma. So, we could assume that a highly aberrant genomic profile results from circulating tumor DNA entering the pool of total cfDNA in maternal plasma. Such examples demonstrate that a routine NIPT test can provide health-related information for the fetus, the mother, and other potential offspring. However, in this context, significant ethical questions that should be discussed arise [36].

CNVs can affect gene expression through complex mechanisms that extend beyond gene dosage effects [37]. Although thousands of miRNA molecules are known, they are only a tenth of nucleotides long. Thus, miRNAs constitute only a tiny portion of the genome, explaining the scarcity of CNV-miRNA overlaps in our cohorts. However, considering the miRNA role in post-transcriptional silencing of gene expression, such CNVs may possess miRNA dosage aberration and thus affect essential physiological processes. On the other hand, most of the biotype overlapping CNVs fell on lncRNAs. Since lncRNA plays important biological roles (e.g., epigenetic regulation of allelic expression, post-transcriptional gene regulation, act as scaffolds for protein complexes or precursors for small non-coding RNAs [38]), their alterations can also affect human metabolism or contribute to the development of pathologies. However, many non-coding RNA sequences remain poorly explored. Thus, it is impossible to reliably conclude the impact of most overlapping CNVs on the physiology of individuals. Nevertheless, knowledge of population genetic studies has significantly influenced our understanding of the genome or clarifying its role in disease development [39–41]. Mapping the regions that can be deleted from the human genome without apparent phenotypic consequences is greatly beneficial for interpreting new CNV findings for clinical and research applications [10]. Following the expansion of CNV analysis in clinical laboratories, these resources will be invaluable to researchers, laboratory diagnostics, and clinical geneticists in structural variant classification.

We have shown several differences between populations at the large-scale CNVs; however, our study has several limitations. In the countries that provided samples for this work, NIPT is not implemented into public prenatal care, so patients must pay for the test. Thus, the selection of patients was not random but preferred individuals who could afford the test. Another limitation may be that only women of reproductive age undergo the test. However, for maternal CNVs, in this study, we only considered highly reliable findings representing germline variants. On the other hand, somatic variants known to accumulate with age form only a minimal fraction of sequencing reads that were excluded from the analyses. Thus, the age limitation resulting from the reproductive capacity of the patients should not affect our findings. Maternal CNVs are detected as per the study in [18], which used non-mixed samples. Precise evaluation of maternal CNV detection limits, particularly in setups with high placental content (i.e., lower maternal content), is lacking. However, such cases are currently rare and excluded from the study. Compared populations were closely related geographically so that the differences could be blurred due to genetic crosses between populations over the years. However, the population comparison in this work serves mainly to demonstrate the usability of the presented approach. At the same time, a much greater benefit is the contribution to the overall knowledge of the Central Europe genome (e.g., the 1 + Million Genomes initiative). Despite the effort for consistency between the laboratories that provided us with data, we cannot rule out some differences in sample manipulation that are important factors affecting the cfDNA analysis [42]. However, these should not affect maternal CNV representations since our method has provided high robustness and reliability for such a purpose [18]. The samples obtained within the laboratory from the studied populations should represent, to some extent, the structure of the population of interest. However, samples from different ethnic groups could also be included; thus, the percentage of variability in individual populations could be increased. So, the information on the ethnicity of patients undergoing the routine test could add value to further such population studies. Moreover, the data are subject to anonymization with no information on the health status of the individual, so we could not relate a patient phenotype to the supposed consequences of CNVs. If the patients were asked to provide at least basic anamnestic and demographic data, it could help add valuable insights into ambiguous variants.

## Conclusion

Our results suggest that the reanalysis of sequencing data from routine low-coverage WGS can potentially obtain population frequencies of larger-scale CNV with no need for additional funds for laboratory sample processing. This offers significant potential for cost-effective expansion of our understanding of population CNVs. While the proposed method was compared with the standard arrayCGH procedure, further verification of this approach would be beneficial.

We conclude that basic anamnestic and demographic data subjected to anonymization could significantly increase the value of such population studies and add valuable insights to support the classification of ambiguous variants. Nevertheless, this approach can provide information to help laboratory diagnosticians and clinical geneticists interpret large-scale CNV.

### Electronic supplementary material

Below is the link to the electronic supplementary material.

Supplementary Material 1

Supplementary Material 2

Supplementary Material 3

## Data Availability

The datasets for this study (both input files, scripts, and output files) can be found in the GitHub: https://github.com/marcelTBI/CNV_population_study.
