# Supplementary material for: Understanding genetic variability: exploring large-scale copy number variants through non-invasive prenatal testing in European populations
Source: BMC Genomics. 2024 Apr 15;25:366. doi: 10.1186/s12864-024-10267-5 (PMC11017555; doi:10.1186/s12864-024-10267-5)
Supplement: Supplementary file 1 — Supplementary Material 1 [file 12864_2024_10267_MOESM1_ESM.docx]

Understanding Genetic Variability: Exploring Copy Number Variants through Non-Invasive Prenatal Testing in European Populations

**Zuzana Holesova*, Ondrej Pös, Juraj Gazdarica, Marcel Kucharik, Jaroslav Budis, Michaela Hyblova, Gabriel Minarik, and Tomas Szemes**

*** Correspondence:** Zuzana Holesova: [zuzana.holesova@geneton.sk](mailto:zuzana.holesova@geneton.sk)

# Supplementary Figure and Tables

**
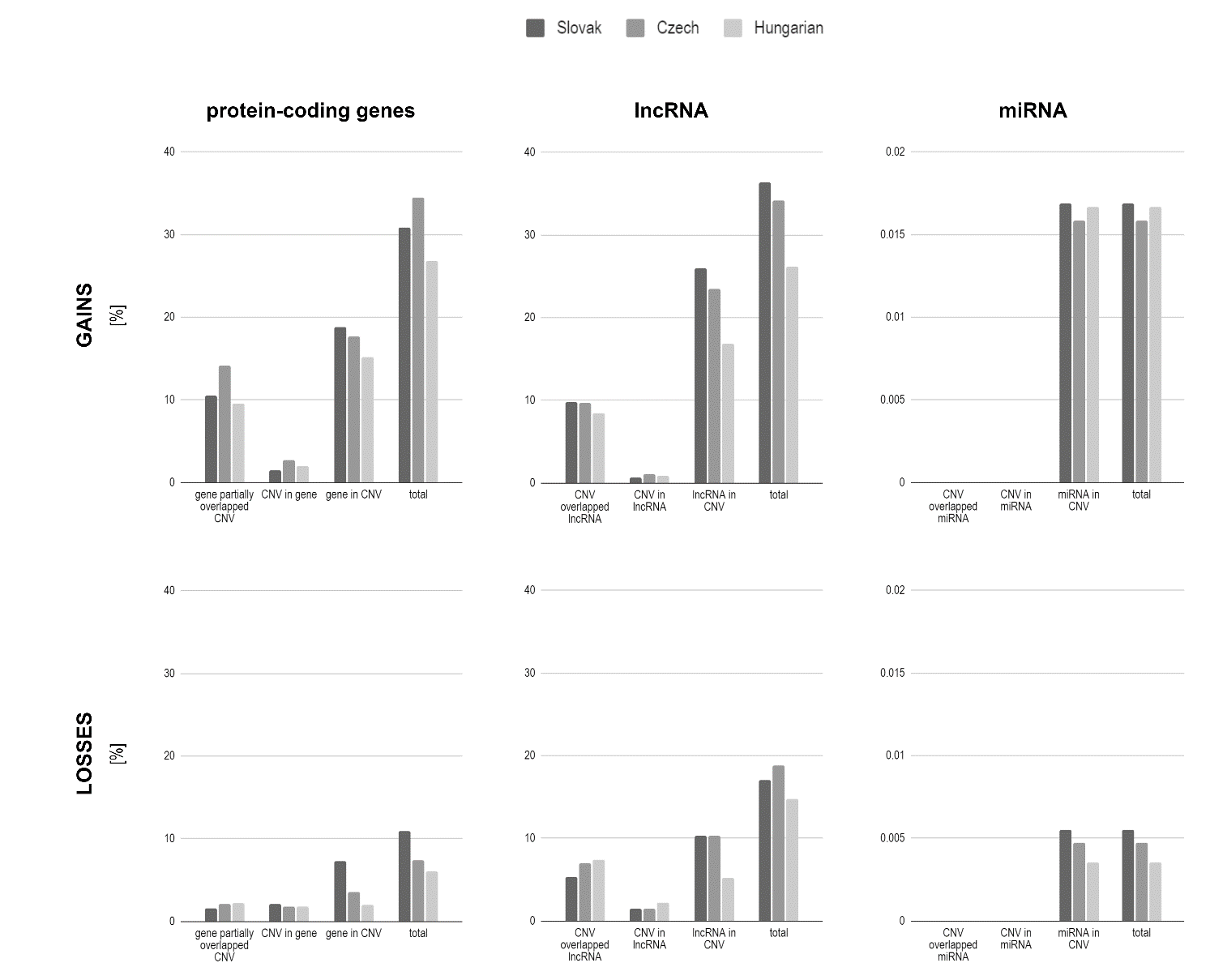
**

**Supplementary Figure 1.** The proportions of CNV overlaps for protein-coding genes, lncRNA, and miRNA calculated separately in the studied populations for gains and losses. **gene** - protein-coding gene, **lncRNA** – long non-coding RNA, **miRNA** – microRNA.

###

**
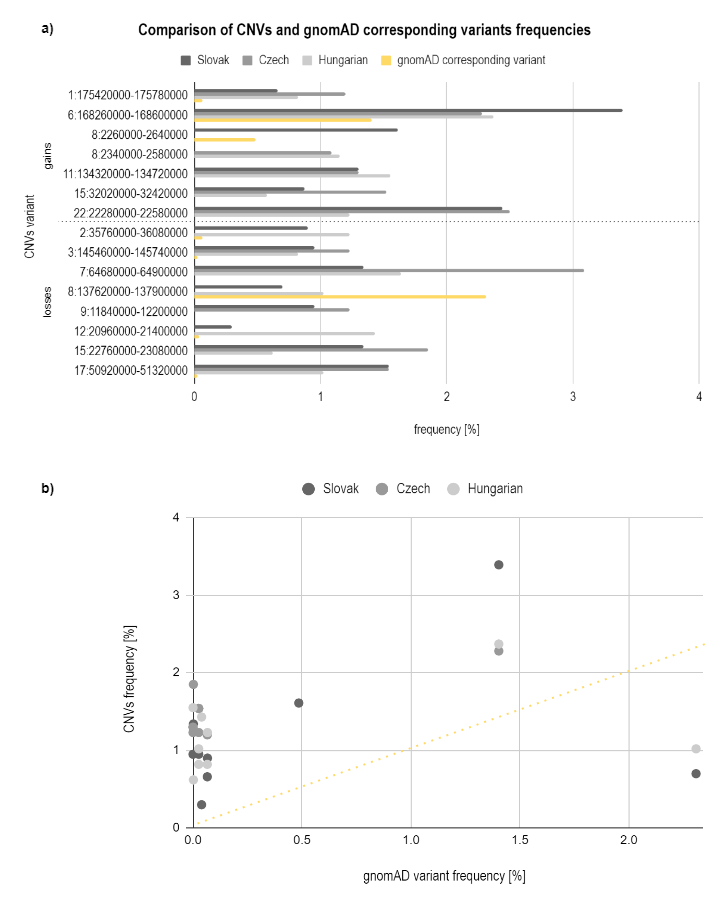
**

### Supplementary Figure 2. Comparison of gain and loss CNVs and gnomAD corresponding variants frequencies.

### Supplementary Table 1. Distribution of gain and loss CNVs on individual chromosomes according to populations.

| **chromosome** | **Slovak** | | | | **Czech** | | | | **Hungarian** | | | |
| --- | --- | --- | --- | --- | --- | --- | --- | --- | --- | --- | --- | --- |
|  | **gains** | | **losses** | | **gains** | | **losses** | | **gains** | | **losses** | |
|  | **count** | **%** | **count** | **%** | **count** | **%** | **count** | **%** | **count** | **%** | **count** | **%** |
| **1** | 143 | 5.55 | 49 | 4.87 | 35 | 7.61 | 10 | 6.17 | 42 | 6.87 | 15 | 6.15 |
| **2** | 134 | 5.2 | 71 | 7.05 | 36 | 7.83 | 4 | 2.47 | 28 | 4.58 | 19 | 7.79 |
| **3** | 123 | 4.77 | 71 | 7.05 | 24 | 5.22 | 15 | 9.26 | 26 | 4.26 | 20 | 8.2 |
| **4** | 101 | 3.92 | 56 | 5.56 | 12 | 2.61 | 9 | 5.56 | 23 | 3.76 | 19 | 7.79 |
| **5** | 88 | 3.41 | 58 | 5.76 | 20 | 4.35 | 10 | 6.17 | 28 | 4.58 | 11 | 4.51 |
| **6** | 300 | 11.64 | 43 | 4.27 | 49 | 10.65 | 6 | 3.7 | 66 | 10.8 | 13 | 5.33 |
| **7** | 147 | 5.7 | 101 | 10.03 | 22 | 4.78 | 23 | 14.2 | 35 | 5.73 | 30 | 12.3 |
| **8** | 194 | 7.53 | 49 | 4.87 | 24 | 5.22 | 10 | 6.17 | 51 | 8.35 | 15 | 6.15 |
| **9** | 72 | 2.79 | 60 | 5.96 | 7 | 1.52 | 15 | 9.26 | 12 | 1.96 | 14 | 5.74 |
| **10** | 65 | 2.52 | 48 | 4.77 | 8 | 1.74 | 9 | 5.56 | 16 | 2.62 | 10 | 4.1 |
| **11** | 146 | 5.66 | 21 | 2.09 | 35 | 7.61 | 4 | 2.47 | 45 | 7.36 | 5 | 2.05 |
| **12** | 120 | 4.65 | 42 | 4.17 | 17 | 3.7 | 3 | 1.85 | 42 | 6.87 | 14 | 5.74 |
| **13** | 47 | 1.82 | 32 | 3.18 | 2 | 0.43 | 5 | 3.09 | 2 | 0.33 | 9 | 3.69 |
| **14** | 62 | 2.4 | 21 | 2.09 | 8 | 1.74 | 1 | 0.62 | 10 | 1.64 | 4 | 1.64 |
| **15** | 143 | 5.55 | 38 | 3.77 | 29 | 6.3 | 7 | 4.32 | 22 | 3.6 | 7 | 2.87 |
| **16** | 56 | 2.17 | 18 | 1.79 | 13 | 2.83 | 3 | 1.85 | 14 | 2.29 | 4 | 1.64 |
| **17** | 51 | 1.98 | 44 | 4.37 | 14 | 3.04 | 7 | 4.32 | 12 | 1.96 | 8 | 3.28 |
| **18** | 28 | 1.09 | 22 | 2.18 | 9 | 1.96 | 1 | 0.62 | 8 | 1.31 | 1 | 0.41 |
| **19** | 44 | 1.71 | 4 | 0.4 | 10 | 2.17 | 0 | 0 | 17 | 2.78 | 1 | 0.41 |
| **20** | 34 | 1.32 | 21 | 2.09 | 7 | 1.52 | 1 | 0.62 | 9 | 1.47 | 5 | 2.05 |
| **21** | 14 | 0.54 | 10 | 0.99 | 3 | 0.65 | 0 | 0 | 2 | 0.33 | 0 | 0 |
| **22** | 172 | 6.67 | 23 | 2.28 | 31 | 6.74 | 1 | 0.62 | 25 | 4.09 | 4 | 1.64 |
| **X** | 294 | 11.4 | 105 | 10.43 | 45 | 9.78 | 18 | 11.11 | 76 | 12.44 | 16 | 6.56 |

###

### Supplementary Table 2. Length range distribution of gain and loss CNVs according to populations.

| **length range** [kbp] | **Slovak** | | | | **Czech** | | | | **Hungarian** | | | |
| --- | --- | --- | --- | --- | --- | --- | --- | --- | --- | --- | --- | --- |
|  | **gains** | | **losses** | | **gains** | | **losses** | | **gains** | | **losses** | |
|  | **count** | **%** | **count** | **%** | **count** | **%** | **count** | **%** | **count** | **%** | **count** | **%** |
| 200-300 | 1068 | 41.43 | 497 | 49.35 | 165 | 35.87 | 79 | 48.77 | 204 | 33.39 | 130 | 53.28 |
| 300-400 | 533 | 20.67 | 243 | 24.13 | 107 | 23.26 | 36 | 22.22 | 130 | 21.28 | 51 | 20.90 |
| 400-500 | 318 | 12.34 | 99 | 9.83 | 66 | 14.35 | 12 | 7.41 | 88 | 14.40 | 27 | 11.07 |
| 500-600 | 151 | 5.86 | 44 | 4.37 | 34 | 7.39 | 8 | 4.94 | 40 | 6.55 | 5 | 2.05 |
| 600-700 | 125 | 4.85 | 23 | 2.28 | 15 | 3.26 | 8 | 4.94 | 31 | 5.07 | 6 | 2.46 |
| 700-800 | 90 | 3.49 | 18 | 1.79 | 19 | 4.13 | 2 | 1.23 | 13 | 2.13 | 11 | 4.51 |
| 800-900 | 58 | 2.25 | 16 | 1.59 | 9 | 1.96 | 3 | 1.85 | 15 | 2.45 | 4 | 1.64 |
| 900-1000 | 34 | 1.32 | 6 | 0.60 | 9 | 1.96 | 3 | 1.85 | 13 | 2.13 | 2 | 0.82 |
| 1000-1100 | 28 | 1.09 | 8 | 0.79 | 3 | 0.65 | 1 | 0.62 | 10 | 1.64 | 1 | 0.41 |
| 1100-1200 | 21 | 0.81 | 6 | 0.60 | 3 | 0.65 | 3 | 1.85 | 4 | 0.65 | 1 | 0.41 |
| 1200-1300 | 24 | 0.93 | 2 | 0.20 | 7 | 1.52 | 0 | 0.00 | 8 | 1.31 | 2 | 0.82 |
| 1300-1400 | 14 | 0.54 | 4 | 0.40 | 2 | 0.43 | 0 | 0.00 | 2 | 0.33 | 1 | 0.41 |
| 1400-1500 | 14 | 0.54 | 2 | 0.20 | 1 | 0.22 | 2 | 1.23 | 3 | 0.49 | 1 | 0.41 |
| 1500-1600 | 5 | 0.19 | 3 | 0.30 | 2 | 0.43 | 0 | 0.00 | 2 | 0.33 | 0 | 0.00 |
| 1600-1700 | 41 | 1.59 | 10 | 0.99 | 6 | 1.30 | 3 | 1.85 | 8 | 1.31 | 2 | 0.82 |
| 1700-1800 | 5 | 0.19 | 1 | 0.10 | 1 | 0.22 | 0 | 0.00 | 3 | 0.49 | 0 | 0.00 |
| 1800-1900 | 3 | 0.12 | 0 | 0.00 | 0 | 0.00 | 0 | 0.00 | 1 | 0.16 | 0 | 0.00 |
| 1900-2000 | 2 | 0.08 | 4 | 0.40 | 1 | 0.22 | 1 | 0.62 | 1 | 0.16 | 0 | 0.00 |
| 2000-3000 | 13 | 0.50 | 5 | 0.50 | 6 | 1.30 | 1 | 0.62 | 3 | 0.49 | 0 | 0.00 |
| 3000-4000 | 3 | 0.12 | 0 | 0.00 | 2 | 0.43 | 0 | 0.00 | 23 | 3.76 | 0 | 0.00 |
| 4000-5000 | 3 | 0.12 | 2 | 0.20 | 0 | 0.00 | 0 | 0.00 | 7 | 1.15 | 0 | 0.00 |
| 5000-6000 | 11 | 0.43 | 2 | 0.20 | 2 | 0.43 | 0 | 0.00 | 2 | 0.33 | 0 | 0.00 |
| over 6000 | 14 | 0.54 | 12 | 1.19 | 0 | 0.00 | 0 | 0.00 | 0 | 0.00 | 0 | 0.00 |

###

### Supplementary Table 3. CNVs with allelic frequency ≥ 1 % in at least one population and comparison of their frequencies between populations.

|  | **CNV position** | **p-value** | | Variant frequency in the population [%]: | | |
| --- | --- | --- | --- | --- | --- | --- |
|  |  | **all pop** | **2 pop** | **Slovak** | **Czech** | **Hungarian** |
| gains | 1:175420000-175780000 | 0.2115 |  | 0.66 | **1.2** | 0.82 |
|  | 6:168260000-168600000 | 0.0526 |  | **3.39** | **2.28** | **2.37** |
|  | 8:2260000-2640000 | **2.18x10^-8^** |  | **1.61** | NA | NA |
|  | 8:2340000-2580000 | **2.29x10^-13^** | 0.8977 | NA | **1.09** | **1.15** |
|  | 11:134320000-134720000 | 0.7769 |  | **1.3** | **1.3** | **1.55** |
|  | 15:32020000-32420000 | 0.0633 |  | 0.875 | **1.52** | 0.575 |
|  | 22:22280000-22580000 | 0.0284 |  | **2.44** | **2.5** | **1.23** |
| losses | 2:35760000-36080000 | 0.1579 | 0.4927 | 0.9 | NA | **1.23** |
|  | 3:145460000-145740000 | 0.335 |  | 0.95 | **1.23** | 0.82 |
|  | 7:64680000-64900000 | 0.0631 |  | **1.34** | **3.09** | **1.64** |
|  | 8:137620000-137900000 | 0.2084 | 0.4502 | 0.7 | NA | **1.02** |
|  | 9:11840000-12200000 | 0.0583 | 0.1484 | 0.95 | **1.23** | NA |
|  | 12:20960000-21400000 | **0.0016** | **0.0017** | 0.3 | NA | **1.43** |
|  | 15:22760000-23080000 | 0.2656 |  | **1.34** | **1.85** | 0.62 |
|  | 17:50920000-51320000 | 0.6836 |  | **1.54** | 1.54 | **1.02** |

**all pop** - all population; **2 pop** - 2 populations, in which the given variant was not zero; **bold number** - statistically significant after Bonferroni correction; allelic frequency over 1 %; **NA** - variant was not found in the given population.

### Supplementary Table 4. CNVs with allele frequency > 1 % found in Slovak, Czech and Hungarian populations and gnomAD structural variants with comparable range. Data also include prediction of clinical significance according to the ISV tool [(Gažiová et al., 2022)](https://paperpile.com/c/igq9Xb/1Tblh), automated ACMG guidelines available at <https://genovisio.com> and X-CNV tool [(Zhang et al., 2021)](https://paperpile.com/c/igq9Xb/WX5HT).

|  | **CNV position** | **size** [bp] | **clinical significance** | | | | | **gnomAD structural variants with comparable range** | | | | |
| --- | --- | --- | --- | --- | --- | --- | --- | --- | --- | --- | --- | --- |
|  |  |  | **ISV** | **ACMG** | **X-CNV** | | | **Variant ID** | **position** | **size** [bp] | **consequence** | **allele frequency** [%] (EU) |
|  |  |  |  |  | **MPV score** | | **related genes** |  |  |  |  |  |
| gains | 1:175420000-175780000 | 360000 | B | VUS | B | 0.123 | TNR | DUP_1_2651 | 1:175430116-175761840 | 331724 | partial duplication | 0.06558 |
|  | 6:168260000-168600000 | 340000 | VUS | VUS | VUS | 0.282 | KIF25, AFDN, FRMD1 | DUP_6_19621* | 6:168333440-168597751 | 264311 | copy gain | 1.403 |
|  | 8:2260000-2640000 | 380000 | B | B | B | 0.123 | - | DUP_8_23522* | 8:1704861-2281924 | 328928 | intergenic | 0.4853 |
|  | **8:2340000-2580000** | 240000 | B | B | B | 0.123 | - | - |  |  |  |  |
|  | 11:134320000-134720000 | 400000 | B | B | B | 0.123 | - | DUP_11_33705 | 11:134336900-134686177 | 439449 | copy gain | 0 |
|  | **15:32020000-32420000** | 400000 | VUS | B | LB | 0.154 | OTUD7A, CHRNA7 | - |  |  |  |  |
|  | **22:22280000-22580000** | 300000 | B | VUS | LB | 0.154 | PPM1F, TOP3B | - |  |  |  |  |
| losses | 2:35760000-36080000 | 320000 | B | VUS | B | 0.123 | - | DEL_2_16311 | 2:35817000-36090000 | 273000 | intergenic | 0.0664 |
|  | 3:145460000-145740000 | 280000 | B | VUS | B | 0.123 | - | DEL_3_38017 | 3:145475545-145753483 | 277938 | intergenic | 0.02623 |
|  | **7:64680000-64900000** | 220000 | B | B | B | 0.123 | ZNF92 | - |  |  |  |  |
|  | 8:137620000-137900000 | 280000 | B | B | B | 0.123 | - | DEL_8_97671 | 8:137680282-137862372 | 182090 | intergenic | 2.308 |
|  | 9:11840000-12200000 | 360000 | B | B | B | 0.123 | - | DEL_9_99632- | 9:11868701-12185026 | 316325 | intergenic | 0 |
|  | 12:20960000-21400000 | 440000 | B | VUS | VUS | 0.187 | SLCO1B1, SLCO1B3, AC022335.1, SLCO1B7, AC011604.2 | DEL_12_126286 | 12:21007643-21412242 | 404599 | loss of function | 0.03935 |
|  | 15:22760000-23080000 | 320000 | VUS | VUS | **P** | 0.84 | NIPA2, NIPA1,  CYFIP1, TUBGCP5 | DEL_15_145847* | 15:22748999-23268000 | 519001 | loss of function | 0.001836 |
|  | 17:50920000-51320000 | 400000 | VUS | VUS | B | 0.123 | - | DEL_17_161110 | 17:50951275-51314632 | 363358 | loss of function | 0.02623 |

**B** - benign, **LB** - likely benign, **P** - pathogenic, **VUS** - variant of uncertain significant; **bold CNV position** - no comparable range was found in gnomAD. ***** variant outside the range of 40,000 bp from one side, **ISV** - Interpretation of Structural Variants prediction method. **ACMG** - The American College of Medical Genetics and Genomics classification. **X-CNV** - analysis interface predicts the pathogenicity of CNVs with GRCh37/hg19 Genome Reference. **MPV score** - meta-voting prediction score, the probability that the CNV is pathogenic. Variant allele frequency in the individual population can be found in Supplementary Table 3.

[Gažiová, M., Sládeček, T., Pös, O., Števko, M., Krampl, W., Pös, Z., et al. (2022). Automated prediction of the clinical impact of structural copy number variations. *Sci. Rep.* 12, 555.](http://paperpile.com/b/igq9Xb/1Tblh)

[Zhang, L., Shi, J., Ouyang, J., Zhang, R., Tao, Y., Yuan, D., et al. (2021). X-CNV: genome-wide prediction of the pathogenicity of copy number variations. *Genome Med.* 13, 132.](http://paperpile.com/b/igq9Xb/WX5HT)

### Supplementary Table 5. The proportions of individual CNV-biotype overlaps for genes, lncRNA, and miRNA calculated separately in the studied populations for gains and losses.

| **overlap type** | **Gains** | | | **Losses** | | |
| --- | --- | --- | --- | --- | --- | --- |
|  | **Slovak** | **Czech** | **Hungarian** | **Slovak** | **Czech** | **Hungarian** |
| gene partially  overlapped CNV | 10.51 % | 14.12 % | 9.60 % | 1.56 % | 2.06 % | 2.20 % |
| CNV in gene | 1.50 % | 2.68 % | 2.04 % | 2.10 % | 1.79 % | 1.79 % |
| gene in CNV | 18.77 % | 17.69 % | 15.13 % | 7.25 % | 3.53 % | 2.01 % |
| total | 30.79 % | 34.49 % | 26.77 % | 10.91 % | 7.39 % | 6.00 % |
| lncRNA partially  overlapped CNV | 9.76 % | 9.67 % | 8.46 % | 5.31 % | 7.03 % | 7.37 % |
| CNV in lncRNA | 0.61 % | 1.08 % | 0.88 % | 1.46 % | 1.54 % | 2.21 % |
| lncRNA in CNV | 25.94 % | 23.44 % | 16.79 % | 10.26 % | 10.28 % | 5.18 % |
| total | 36.30 % | 34.18 % | 26.13 % | 17.03 % | 18.84 % | 14.77 % |
| miRNA partially  overlapped CNV | 0 % | 0 % | 0 % | 0 % | 0 % | 0 % |
| CNV in miRNA | 0 % | 0 % | 0 % | 0 % | 0 % | 0 % |
| miRNA in CNV | 0.017 % | 0.016 % | 0.017 % | 0.006 % | 0.005 % | 0.004 % |
| total | 0.017 % | 0.016 % | 0.017 % | 0.006 % | 0.005 % | 0.004 % |

**gene** - protein-coding gene, **lncRNA** - long non-coding RNA, **miRNA** - microRNA.

### 
