# Supplementary figures and images for: Understanding genetic variability: exploring large-scale copy number variants through non-invasive prenatal testing in European populations
Source: BMC Genomics. 2024 Apr 15;25:366. doi: 10.1186/s12864-024-10267-5 (PMC11017555; doi:10.1186/s12864-024-10267-5)

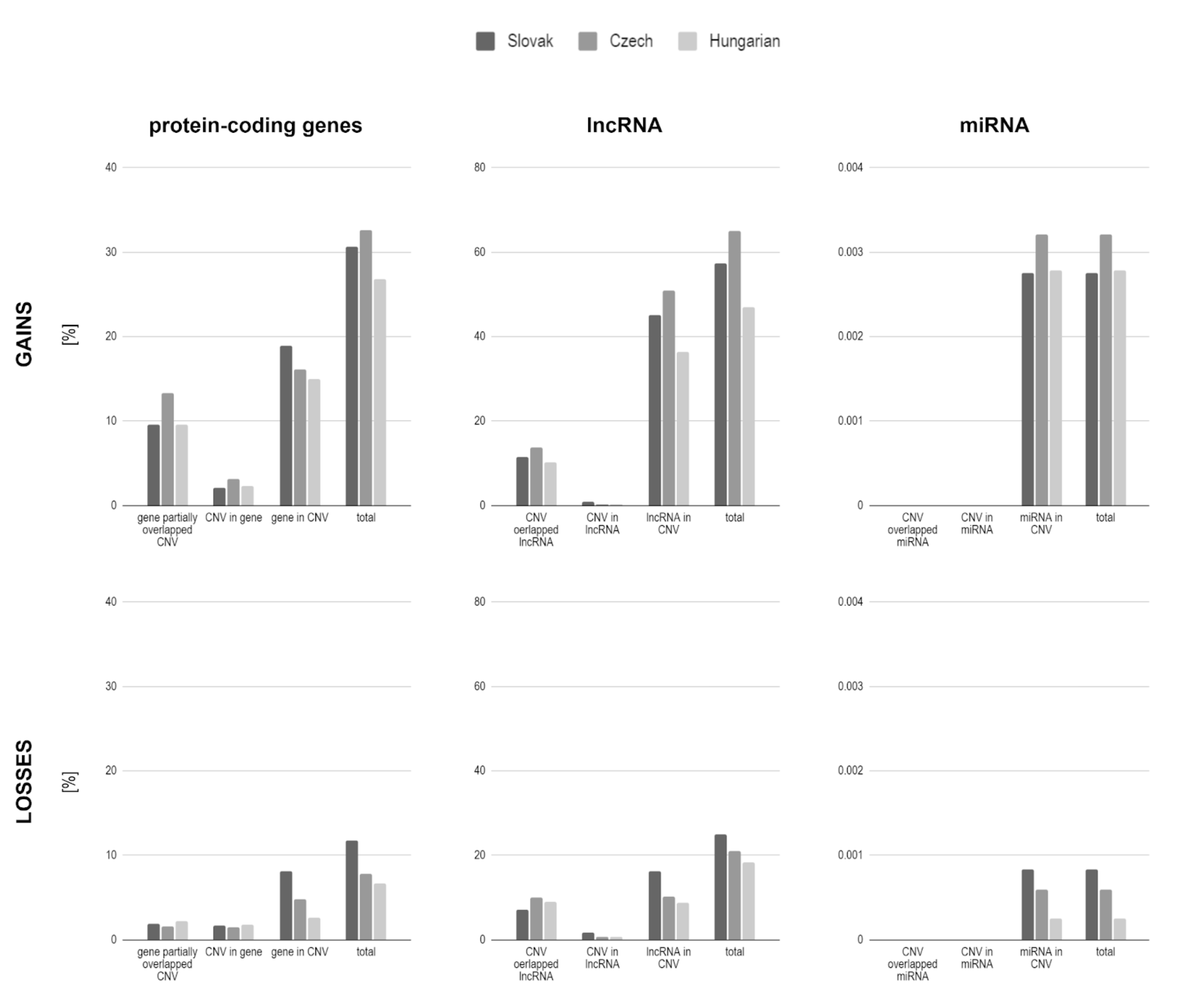

Supplement: Supplementary file 2 — Supplementary Material 2 [file 12864_2024_10267_MOESM2_ESM.png]

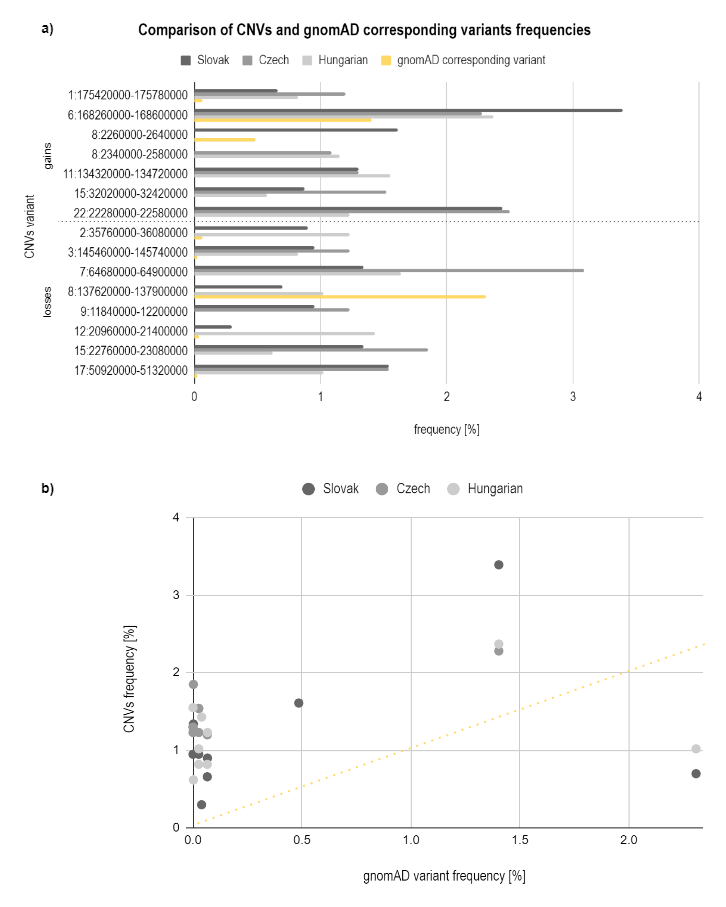

Supplement: Supplementary file 3 — Supplementary Material 3 [file 12864_2024_10267_MOESM3_ESM.png]
